# False-Positive Blood Cultures in Acute Leukemia: An Underrecognized Finding

**DOI:** 10.1155/2018/7090931

**Published:** 2018-01-29

**Authors:** Stamatis Karakonstantis, Ioanna Manika, Maria Vakonaki, Anna Boula

**Affiliations:** ^1^2nd Department of Internal Medicine, General Hospital of Heraklion Venizeleio-Pananeio, Leoforos Knossou, Heraklion, Greece; ^2^Hematology Department, General Hospital of Heraklion Venizeleio-Pananeio, Heraklion, Greece; ^3^Microbiology Department, General Hospital of Heraklion Venizeleio-Pananeio, Heraklion, Greece

## Abstract

The occurrence of false-positive blood cultures in patients with acute myeloid leukemia has been rarely described in the literature. Awareness of this finding is important to avoid unnecessary delays in initiating appropriate cytoreductive therapy. Here, we present the case of a 70-year-old male with acute leukemia and persistently positive blood cultures despite broad-spectrum antibiotic therapy. No source of infection could be found clinically, and no pathogen could be isolated from blood cultures. Inspection of the CO_2_ plots of the positive blood cultures showed a steady linear increase in CO_2_ levels, suggesting false-positive detection by the automated microbial detection system. Cytoreductive therapy was then initiated, and several subsequent blood cultures were negative.

## 1. Introduction

Automated microbial detection systems (AMDSs) are widely used in microbiology laboratories to allow a rapid and automated detection of positive blood cultures. Such systems rely on continuous monitoring, through a colorimetric or fluorescent sensor, of carbon dioxide (CO_2_) produced by growing microorganisms. The algorithm for detection of growth is based on an analysis of the rate of change of CO_2_ concentration [1]. However, about 1–10% of samples processed are flagged positive by AMDSs despite the absence of microorganisms on gram stain and appropriate subcultures [2–4]. Such cases are described as “instrument false-positive” and have been attributed to background CO_2_ production, possibly by white blood cells (WBC) [5].

Instrument false-positive blood cultures in the setting of acute myeloid leukemia (AML) have been rarely described in the literature [6–8]. This finding has been attributed to CO_2_ production by metabolically active blasts, thus leading to a positive signal by AMDSs, but no microorganism is detected on microscopic evaluation and appropriate subcultures. Of note is the recently reported observation that a false-positive blood culture due to leukocytosis shows a steady linear increase in CO_2_ production in contrast to the exponential increase in true-positive blood cultures [8].

## 2. Case Presentation

**Figure 1:**
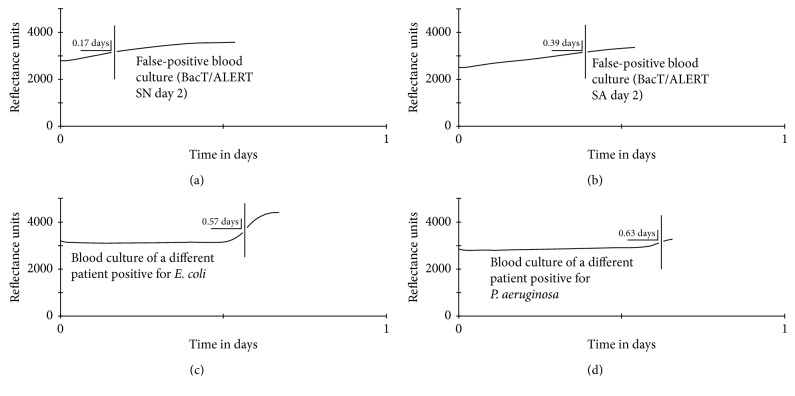
CO_2_ plots (measured with reflectance photometry-BacT/ALERT 3D) of two of our patient's false-positive blood cultures compared to two true-positive blood cultures. The *y*-axis depicts reflectance units and the *x*-axis depicts time in days. The vertical line represents the time that the culture was flagged positive. Note the linear CO_2_ increase of our case's blood cultures. A lag phase, a log phase, and a stationary phase can be seen in both true-positive cultures. In the last example, however, the lag phase is longer and the log phase is shorter, mimicking a linear appearance.

A 70-year-old male with a history of benign prostate hyperplasia and papillary thyroid carcinoma (treated with total thyroidectomy and radioiodine therapy about 6 years ago) presented to the hospital due to asthenia, low-grade fever (37-38°C), and dysuria since about 7 days. The patient was hemodynamically stable. The physical examination and a chest X-ray did not identify a source of infection. His lab tests (including immunophenotyping and karyotype) revealed acute monocytic leukemia (FAB M5) (WBC count = 127 × 10^9^/L) and acute kidney injury. His lab tests less than a month before were normal. Urine and blood cultures were obtained, and he was started on empiric antibiotic therapy with levofloxacin pending culture results. Several blood cultures were flagged positive within 24 hours of incubation (BacT/ALERT® 3D) (Table 1). This combined with the persistence of fever (as high as 38.5°C) led to several changes in the antibiotic treatment regimen (including piperacillin/tazobactam, followed by meropenem-teicoplanin, followed by daptomycin-cefepime) and delayed initiation of AML-specific treatment. However, no microorganisms could be identified on microscopy and appropriate subcultures, and the CO_2_ plot of positive blood cultures was linear (in contrast to the exponential rise of CO_2_ in true-positive cultures) (Figure 1). The patient was started on hydroxyurea (at the 8th day of hospitalization) and tumor lysis syndrome prophylaxis, which led to significant reduction of WBC and improvement of kidney function. Several subsequent blood cultures were negative.

## 3. Discussion

Similar previous cases are summarized in Table 1. Of interest is the observation that in all published cases blood cultures were flagged positive within 24 hours. This is important because a fast time to positivity is often used as an indication of true bacteremia versus contamination [9]. Finally, like the case presented by Khan et al. [8], all false-positive blood cultures in our case showed a linear CO_2_ plot (Figure 1). On the contrary, in the case published by Meessen et al. [7], the growth curve was reported as indistinguishable from true-positive blood cultures, although a description or depiction of these curves was not provided. To further examine this, we reviewed the growth curves of the last 100 positive blood cultures of our lab. All true-positive cultures showed the typical phases of bacterial growth, that is, a lag phase followed by a log phase and then a stationary phase. However, in some cases, we observed a long lag phase and a short, low-slope log phase, mimicking the linear CO_2_ plots of false-positive cultures (Figure 1), which might explain the observation of Meessen et al. [7].

Other factors associated with false-positive cultures include an elevated WBC count [4] and culture bottle overfilling [10], although false-positive cultures are also seen in patients with a normal or even low WBC count [2–4]. Overfilling cannot explain our case. Bottle overfilling most commonly occurs when the direct-draw blood sampling technique is used [10], which is not commonly used in our setting. Furthermore, accidental overfilling of all 4 false-positive blood culture bottles is unlikely. Autolysis of *Streptococcus pneumoniae* is another well-known cause of false-positive blood cultures [11]. This occurs due to production of autolysin by *S. pneumoniae* during its stationary growth phase and can be prevented by early processing of positive culture bottles. *S. pneumoniae* antigen testing has been described as a sensitive method to detect such cases [12] but is not available in our lab. However, this scenario is unlikely in our case because (1) no “chocolatization” of the blood culture medium was noted (99% negative predictive value according to a previous study [13]) and (2) the CO_2_ plot did not show the typical exponential rise of true-positive cultures.

In most cases, a positive signal by AMDSs in the absence of microorganism detection by gram stain and appropriate subcultures represents an “instrument false-positive” rather than the presence of fastidious organisms, as molecular testing for bacterial and fungal DNA failed to identify any microorganism in previous investigations of false-positive cultures [2–4]. However, in the study of Karahan et al. [3], eubacterial PCR was positive in 10 of 104 false-positive BacT/ALERT bottles. Nevertheless, 80% of these cases were positive for skin flora bacteria and could therefore be regarded as contaminants. Performing appropriate subcultures and using appropriate incubation conditions for anaerobic bottles is important to avoid mislabeling positive blood cultures as instrument false-positive. In our case, repeated blood cultures under appropriate incubation conditions and appropriate subcultures failed to identify any microorganism. The persistently positive blood cultures despite a broad-spectrum antimicrobial therapy, and the fact that blood cultures became negative after cytoreduction with hydroxyurea, support our hypothesis of instrument false-positive blood cultures associated with the very elevated peripheral blast number.

The scarcity of reports regarding the occurrence of instrument false-positive blood cultures in acute leukemia is unexpected considering the frequency of this finding (1–10% of all blood cultures) and the high WBC count in acute leukemia. This could represent underrecognition of this finding by clinicians or underreporting. We are not aware of any published paper describing the frequency of false-positive blood cultures in patients with acute leukemia. In our case, a lack of awareness of this phenomenon led to several changes in the antimicrobial regimen and delayed the initiation of appropriate cytoreductive therapy.

In conclusion, knowledge of the association of AML with false-positive blood cultures is important to allow better management of these patients and possibly prevent unnecessary delays in AML-specific treatment. The CO_2_ plot could be useful for early identification of such cases.

## Figures and Tables

**Table 1:** Summary of previous case reports.

	Patient	Blood culture results
Martinez et al. [6]	67-year-old male. 2 months history of asthenia. Fever 37.5–38°C. Acute myelomonocytic leukemia. *WBC count* *=* *150* *×* *10*^*9*^*/L (95% blasts).*	(i) AMDS: BACTEC NR730 and BacT/ALERT.
		(ii) All 6 blood culture bottles were flagged positive: at 18 hours (aerobic, BACTEC NR370), 48 hours (anaerobic BACTEC NR370), 3.3 and 3.4 hours (two anaerobic bottles, BacT/ALERT), and at 12.2 hours (both aerobic bottles, BacT/ALERT).
		(iii) No information on the CO_2_ plot.
Meessen et al. [7]	56-year-old female with a 3-week history of malaise and fever (39°C). Diagnosed with AML. *WBC count* *=* *446 × 10^9^/L (100% blasts).*	(i) AMDS: BACTEC 9240.
		(ii) Two culture bottles (BACTEC^PLUS^/F, aerobic, and anaerobic bottle) (from a total of 4 sets) flagged positive at 19.1 (aerobic) and 4.3 (anaerobic) hours.
		(iii) The shapes of the growth curves were indistinguishable from those observed during bacterial growth.
Khan et al. [8]	67-year-old male, with relapsed/refractory AML. Presentation with fever, fatigue, and mucositis. The patient was on protocol therapy with azacitidine and nivolumab for relapsed disease. Maximum recorded fever 38.6°C. A chest computed tomography showed no evidence of infection. *WBC count* *=* *37 × 10^9^/L (87% blasts).*	(i) AMDSs: BACTEC FX.
		(ii) The single blood culture obtained (BACTEC^PLUS^/F, aerobic culture bottle) was flagged positive at 14 hours.
		(iii) Linear CO_2_ plot.
Our case	70-year-old male with asthenia and low-grade fever. Maximum recorded fever 38.5. Diagnosed with acute monocytic leukemia (FAB M5). *WBC count* *=* *127 × 10^9^/L (82% blasts)*.	(i) AMDS: BacT/ALERT 3D.
		(ii) 4 false-positive blood culture bottles. BacT/ALERT SA (aerobic culture taken at day 1) flagged positive at 11.3 hours, BacT/ALERT SA (aerobic culture taken at day 2) flagged positive at 9.4 hours, BacT/ALERT SN (anaerobic culture taken at day 2) flagged positive at 4.1 hours, and BacT/ALERT FN Plus (anaerobic culture taken at day 5) flagged positive at 2.2 hours. 7 subsequent blood cultures were negative.
		(iii) Linear CO_2_ plot in all four false-positive cultures.

AMDS: automated microbial detection system; AML: acute myeloid leukemia; WBC: white blood cells.
